# Supplementary material for: Endocrine Signals Altered by Heat Stress Impact Dairy Cow Mammary Cellular Processes at Different Stages of the Dry Period
Source: Animals (Basel). 2021 Feb 21;11(2):563. doi: 10.3390/ani11020563 (PMC7930950; doi:10.3390/ani11020563)
Supplement: Supplementary file 1 [file animals-11-00563-s001.pdf]

animals

MDPI

Supplementary Material

Endocrine Signals Altered by Heat Stress Impact Dairy Cow Mammary Cellular Processes at Different Stages of the Dry Period

Véronique Ouellet [1], João Negrao [1,2], Amy L. Skibiel [1,†], Valerie A. Lantigua [1], Thiago F. Fabris [1], Marcela G. Marrero [1], Bethany Dado-Senn [1,‡], Jimena Laporta [1,‡] and Geoffrey E. Dahl [1,*]

[1] Department of Animal Sciences, University of Florida, Gainesville, FL, 32611, USA; veronique.ouellet.6@ulaval.ca (V.O.); jnegrao@usp.br (J.N.); askibiel@uidaho.edu (A.L.S.); vlantigua@ufl.edu (V.A.L.); tfabris89@gmail.com (T.F.F.); marcela.marrero@ufl.edu (M.G.M.); bethanydado@ufl.edu (B.D.-S.); jlaporta@wisc.edu (J.L.)

[2] Department of Basic Sciences, Faculty of Animal Science and Food Engineering, University of Sao Paulo, Pirassununga, SP, USA

* Correspondence: gdahl@ufl.edu; Tel.: +1-352-294-6980; Fax: +1-352-392-5595

† Current address: Department of Animal and Veterinary Science, University of Idaho, Moscow, ID 83844, USA.

‡ Current address: Department of Animal and Dairy Sciences, University of Wisconsin Madison, WI 53706, USA.

Citation: Ouellet, V.; Negrao, J.; Skibiel, A.L.; Lantigua, V.A.; Fabris, T.F.; Marrero, M.G.; Dado-Senn, B.; Laporta, J.; Dahl, G.E. Endocrine Signals Altered by Heat Stress Impact Dairy Cow Mammary Cellular Processes at Different Stages of the Dry Period. *Animals* **2021**, *11*, 563. https://doi.org/10.3390/ani11020563

Academic Editor: Jong-Eun Park and Himansu Kumar

Received: 12 January 2021
Accepted: 15 February 2021
Published: 21 February 2021

Publisher's Note: MDPI stays neutral with regard to jurisdictional claims in published maps and institutional affiliations.

Copyright: © 2021 by the authors. Submitted for possible open access publication under the terms and conditions of the Creative Commons Attribution (CC BY) license (http://creativecommons.org/licenses/by/4.0/).

Table S1. Primers used in quantitative real-Time PCR assay gene expression.

Gene	Primer	Sequence (5′-3′)	Accession No.
ACTB	Forward<br>Reverse	ACACCTTCTACAATGAGCTG<br>CTGCTTGCTGATCCACATCT	NM_173979.3
AIFM1	Forward<br>Reverse	GGCTTCCTTGGTAGCGAACTGG<br>GTCCAGTTGCTGAGGTATTCGG	NM_001192984.1
AKT1	Forward<br>Reverse	CTGCACAAGCGAGGTGAGTA<br>GAGAAGTTGTTGAGGGGCGA	NM_173986.2
AKT2	Forward<br>Reverse	CATCCTCATGGAAGAGATCCGC<br>GAGGAAGAACCTGTGCTCCATG	NM_001206146.1
AMBRA1	Forward<br>Reverse	CTCTTCCTCAGACAACCAGGGT<br>TCCAAGCGAAGGTGCAGACATC	NM_001034522.2
APAF1	Forward<br>Reverse	GCCAAGCAGGAGGTCGATAATG<br>GACCATCCTCAGAAAAGCAGGC	NM_001191507.1
ATG3	Forward<br>Reverse	GGTTGTTCGGCTATGATGAG<br>GGGAGATGAGGGTGATTTTC	NM_001075364.1
ATG5	Forward<br>Reverse	TTTGAATATGAAGGCACACC<br>TGTAAACCCATCCAGAGTTG	NM_001034579.2
ATG7	Forward<br>Reverse	CGTTGCCCACAGCATCATCTTC<br>CACTGAGGTTCACCATCCTTGG	NM_001083795.2
BACH2	Forward<br>Reverse	CTGCCGCAAAAGGAAACTGGAC<br>GGAAAGGCAGGAGAAGTTGTCC	XM_024996978.1
BAD	Forward<br>Reverse	TCCCAGAGTTTGAGCAGAGTG<br>TTAGCCAGTGCTTGCTGAGAC	NM_001035459.2
BAX	Forward<br>Reverse	GGGTTGTCGCCCTTTTCTACTT<br>CGCTCTCGAAGGAAGTCCAATG	NM_173894.1
BCL2	Forward<br>Reverse	ACAGCATCGCCCTGTGGATG<br>GTGCCTTCAGAGACAGCCAG	NM_001166486.1
BECN1	Forward<br>Reverse	GATGGAATAGGAACCACCAC<br>AGTTGAGAAAGGCGAGACAC	NM_001033627.2
BNIP3	Forward<br>Reverse	TCAGCATGAGGAACACGAGCGT<br>GAGGTTGTCAGACGCCTTCCAA	NM_001076366.1
BRAF	Forward<br>Reverse	AACGAGACCGATCCTCATCAGC<br>GGTAGCAGACAAACCTGTGGTTG	XM_024991194.1
CASP3	Forward<br>Reverse	TGAAATACGAAGTCAGGATTAAA<br>GTCCGTTGGTTCCAAAAATG	NM_001077840.1
CASP8	Forward<br>Reverse	TTTAGCATAGCACGGAAGCA<br>TATCCAAAGCGTCTGCATCA	NM_001045970.2
CASP9	Forward<br>Reverse	GTTTGAGGACCTTCGACCAGCT<br>CAACGTACCAGGAGCCACTCTT	NM_001205504.1
CD4	Forward<br>Reverse	CGGAATTCGCCCCTTCTCCAGCAGAT<br>CCGCTCGAGGATGGCAACACAAAGC	NM_001103225.1
CDKN1B	Forward<br>Reverse	TGTCAAACGTGCGAGTGTCTA<br>CTCTGCAGTGCTTCTCCAAGT	NM_001100346.1
CREB1	Forward<br>Reverse	GACCACTGATGGACAGCAGATC<br>GAGGATGCCATAACAACTCCAGG	NM_174285.1
CSN2	Forward<br>Reverse	GCTATGGCTCCTAAGCACAAAGA<br>GGAAACATGACAGTTGGAGGAAG	BC_111172.1
CXCL2	Forward<br>Reverse	GGCAGAAAGCTTGTCTCAACCC<br>CTCCTTCAGGAACAGCCACCAA	NM_174299.3
CXCL8	Forward<br>Reverse	GAGAGTGATTGAGAGTGGACCAC<br>CACAACCCTCTGCACCCAGTTT	NM_173925.2
DACT2	Forward<br>Reverse	CTACACCAGGAGCGACTCAGAG<br>ACTCACGGTCTCCGAATCGGTT	XM_024997160.1
EGF	Forward<br>Reverse	TGCGATGCCAAGCAGTCTGTGA<br>GCATAGCCCAATCTGAGAACCAC	XM_024993778.1

ESR1	Forward<br>Reverse	GCTTACTGACCAACCTGGCAGA<br>GGATCTCTAGCCAGGCACATTC	NM_001001443.1
ESR2	Forward<br>Reverse	ATGGAGTCTGGTCGTGTGAAGG<br>TAACACTTCCGAAGTCGGCAGG	NM_174051.3
Fas	Forward<br>Reverse	GGACCCAGAATACCAAGTGCAG<br>GTTGCTGGTGAGTGTGCATTCC	NM_174662.2
FASLG	Forward<br>Reverse	GGTTCTGGTTGCCTTGGTAGGA<br>CTGTGTGCATCTGGCTGGTAGA	NM_001098859.2
FOXO3	Forward<br>Reverse	TCTACGAGTGGATGGTGCGTTG<br>CTCTTGCCAGTTCCCTCATTCTG	NM_001206083.1
GAPDH	Forward<br>Reverse	TGACCCCTTCATTGACCTTC<br>TACTCAGCACCAGCATCACC	NM_001034034.2
GPER1	Forward<br>Reverse	TTCCGCGAGAAGATGACCATCC<br>TAGTACCGCTCGTGCAGGTTGA	XM_024985174.1
GRB2	Forward<br>Reverse	GAAATGCTTAGCAAACAGCGGCA<br>TCCATCTCGGAGCACCTTGAAG	NM_001034630.1
HPRT1	Forward<br>Reverse	CATTATGCTGAGGATTTGGAAAGG<br>CTTGAGCACACAGAGGGCTACA	NM_001034035.2
HRAS	Forward<br>Reverse	ACGCACTGTGGAATCTCGGCAG<br>TCACGCACCAACGTGTAGAAGG	NM_001242347.1
HSD17B6	Forward<br>Reverse	CCAGCATTCTGGGAAGAGTTGC<br>CCGTTCTGAAGTAGCCAGGTTC	NM_001035395.2
HSF1	Forward<br>Reverse	CAGCTGATGAAGGGGAAGCA<br>TGGATGAGCTTGTTGACGACT	NM_001076809.1
HSP90AA1	Forward<br>Reverse	TCTGCCTCTGGTGATGAGATGG<br>CGTTCCACAAAGGCTGAGTTAGC	NM_001012670.2
HSPA1A	Forward<br>Reverse	ACCTTCGACGTGTCCATCCTGA<br>TCCTCCACGAAGTGGTTCACCA	NM_203322.3
IGFBP3	Forward<br>Reverse	CGCTACAAAGTTGACTACGAGTC<br>GTCTTCCATTTCTCTACGGCAGG	NM_174556.1
IGFBP5	Forward<br>Reverse	CGTGCTGTGTACCTGCCCAATT<br>ACTTGTCCACGCACCAGCAGAT	NM_001105327.2
IL1A	Forward<br>Reverse	TGTATGTGACTGCCCAAGATGAAG<br>AGAGGAGGTTGGTCTCACTACC	NM_174092.1
IL1B	Forward<br>Reverse	CCACAGACCTTCCAGGAGAATG<br>GTGCAGTTCAGTGATCGTACAGG	NM_174093.1
IL-6	Forward<br>Reverse	AGACAGCCACTCACCTCTTCAG<br>TTCTGCCAGTGCCTCTTTGCTG	NM_173923.2
INSIG1	Forward<br>Reverse	TTTTCTCAGGAGGCGTCACGGT<br>TCCTTGCTCTCAGAATCGGTGG	NM_001077909.1
LBP	Forward<br>Reverse	TCAGCATCTCCGACTCCTCCAT<br>CAGGAGGTTGACCGAAATGCTG	NM_001038674.2
LCA5	Forward<br>Reverse	ATCGTCTGCCAAAGTCCTCTCC<br>CTTCTGGCTTGAAGTCTTCCATG	NM_001195068.1
LPL	Forward<br>Reverse	CTGCTGGCATTGCAGGAAGTCT<br>CATCAGGAGAAAGACGACTCGG	NM_001075120.1
MAP1LC3A	Forward<br>Reverse	GCTACAAGGGTGAGAAGCAGCT<br>CTGGTTCACCAGCAGGAAGAAG	NM_001046175.1
MAP1LC3B	Forward<br>Reverse	GAGAAGCAGCTTCCTGTTCTGG<br>GTGTCCGTTCACCAACAGGAAG	NM_001001169.1
MAPK1	Forward<br>Reverse	ACACCAACCTCTCGTACATCGG<br>TGGCAGTAGGTCTGGTGCTCAA	NM_175793.2
MAPK10	Forward<br>Reverse	GTGTGGAAGTGGGAGACTCAAC<br>GTCAAGGACAGCATCATACGCG	NM_001318200.1
MAPK14	Forward<br>Reverse	GAGCGTTACCAGAACCTGTCTC<br>AGTAACCGCAGTTCTCTGTAGGT	NM_001102174.1

MAP2K1	Forward<br>Reverse	GGTGTTCAAGGTCTCCCACAAG<br>CCACGATGTACGGAGAGTTGCA	NM_001130752.1
MAPK3	Forward<br>Reverse	TGGCAAGCACTACCTGGATCAG<br>GCAGAGACTGTAGGTAGTTTCGG	NM_001110018.1
mTOR	Forward<br>Reverse	GGTTAACACCAAGCAGGTTCAT<br>GTATGTCGCACTGGACACCA	XM_002694043.6
NFKB1	Forward<br>Reverse	GCAGCACTACTTCTTGACCACC<br>TCTGCTCCTGAGCATTGACGTC	NM_001076409.1
PCNA	Forward<br>Reverse	CCTTGGTGCAGCTAACCCTT<br>TTGGACATGCTTGGTGAGGTT	NM_001034494.1
PDK1	Forward<br>Reverse	CATGTCACGCTGGGTAATGAGG<br>CTCAACACGAGGTCTTGGTGCA	NM_001205957.1
PIK3CB	Forward<br>Reverse	GGTAATCGGAGGATAGGGCAGT<br>CGGCAGTATGCTTCAAGGATGAC	NM_001206047.1
PIK3R2	Forward<br>Reverse	ATGGCACCTTCCTAGTCCGAGA<br>CTCTGAGAAGCCATAGTGCCCA	NM_174576.2
PRKACA	Forward<br>Reverse	CCACTATGCCATGAAGATCCTCG<br>CGAGTTTGACGAGGAACGGAAAG	NM_174584.2
PRL	Forward<br>Reverse	GAGGAGCAAACCAAACGGCTTC<br>AAGGCGAGACTCTTCATCAGCC	NM_173953.2
PRLR (long)	Forward<br>Reverse	AAGGCCATGTGGAAGATTTG<br>GATGACTGTGAGGACCAGCA	NM_001039726.2
PRLR (short)	Forward<br>Reverse	AGGTGACACTATAGAATAAGCAAC<br>GTACGACTCACTATAGGGAAAGGC	NM_174155.3
PTEN	Forward<br>Reverse	TGAGTTCCCTCAGCCGTTACCT<br>GAGGTTTCCTCTGGTCCTGGTA	NM_001319898.1
PTGES	Forward<br>Reverse	GAGGATGCCCTGAGACACGGA<br>CCAGAAAGGAGTAGACGAAGCC	NM_174443.2
RAC1	Forward<br>Reverse	CGGTGAATCTGGGCTTATGGGA<br>GGAGGTTATATCCTTACCGTACG	NM_174163.2
RHEB	Forward<br>Reverse	CTATCTTTCCTCAGACATACTCCA<br>CACCATATCCAACAATTTGCCATG	NM_001031764.2
RHOA	Forward<br>Reverse	TCTGTCCCAACGTGCCCATCAT<br>CTGCCTTCTTCAGGTTTCACCG	NM_176645.3
RSP9	Forward<br>Reverse	GGAGACCCTTCGAGAAGTCC<br>CTTTCTCATCCAGCGTCAGC	NM_001101152.2
SOCS2	Forward<br>Reverse	GGGAACTCAGTCACACAGGTTGG<br>TGTTAGTAGGTAGTCTGAATGCGAAC	NM_177523.2
SOCS3	Forward<br>Reverse	AGAAGATCCCTCTGGTGTTGAGC<br>GTGACTTTCTCGTAGGAGTCCAGG	NM_174466.2
SQSTM1	Forward<br>Reverse	TGTGTAGCGTCTGCGAGGGAAA<br>AGTGTCCGTGTTTCACCTTCCG	NM_176641.1
STAT1	Forward<br>Reverse	ATGGCAGTCTGGCGGCTGAATT<br>CCAAACCAGGCTGGCACAATTG	NM_00107790.1
STAT3	Forward<br>Reverse	CTTTGAGACCGAGGTGTATCACC<br>GGTCAGCATGTTGTACCACAGG	NM_001012671.2
STAT5A	Forward<br>Reverse	GAAACATCACAAGCCCCATT<br>TGAAGCGCAACAAGAAGGTA	NM_001012673.1
STAT5B	Forward<br>Reverse	GTTTCGTGAACAAGCAACAGGC<br>GGCATCAGATTCCAAAACATTCTTTCC	NM_174617.4
TEAD4	Forward<br>Reverse	GAAGGTCTGCTCTTTCGGCAAG<br>GAGGTGCTTGAGCTTGTGGATG	XM_010805630.3
TEKT3	Forward<br>Reverse	GGACAAGACTGCCTTCCTGAAG<br>GTCTGGATGGTGTCGTCAACCT	NM_001099019.2
TFEB	Forward<br>Reverse	CCTGGAGATGACCAACAAGCAG<br>TAGGCAGCTCCTGCTTCACCAC	NM_001205666.1

TGFB1	Forward<br>Reverse	CCTGAGCCAGAGGGGACTAC<br>GCTCGGACGTGTTGAAGAAC	NM_001166068.1
TGFB3	Forward<br>Reverse	CTAAGCGGAATGAGCAGAGGATC<br>TCTCAACAGCCACTCACGCACA	NM_001101183.1
TLR4	Forward<br>Reverse	ACTGCAGCTTCAACCGTATC<br>TAAAGGCTCTGCACACATCA	NM_174198.6
TNF	Forward<br>Reverse	TAACAAGCCGGTAGCCCACG<br>TCTTGATGGCAGACAGGATG	NM_173966.3
TNFR1	Forward<br>Reverse	CCGCTTCAGAAAACCACCTCAG<br>ATGCCGGTACTGGTTCTTCCTG	NM_174674.2
ULK1	Forward<br>Reverse	GCAAGGACTCTTCCTGTGACAC<br>CCACTGCACATCAGGCTGTCTG	NM_001205927.1
VEGFA	Forward<br>Reverse	TTGCCTTGCTGCTCTACCTCCA<br>GATGGCAGTAGCTGCGCTGATA	NM_001316956.1
WIF1	Forward<br>Reverse	GGTGCCGAAATGGAGGCTTTTG<br>GATGCAGAAACCAGGAGTCACAC	NM_001075996.1
